# Editor's Letter-Box

**Published:** 1894-07-21

**Authors:** 


### EDITOR'S LETTER-BOX.

[Our correspondents are reminded that proxility is a great bar to publication, and that brevity of style and conciseness of statement greatly facilitate early insertion.]

Mr. Washington Epps, L.R.C.P., M.R.C.S., writes to us from 89, Great Russell Street, Bloomsbury, W.C., as follows: "If any of your readers are interested to see particulars of some of the cases of typhus mentioned in The Hospital for June 2nd, under "Typhus Fever, and Difficulties in Diagnosis," they will find a full report, by Drs. Galley Blackley and Byres Mair, of nine of the cases, with the temperature charts of each, in Vol. 2, p. 91, of the "London Homœopathic Reports." These Reports can be obtained at the hospital, Great Ormond Street, W.C.

